# Pharmacological targeting of BET proteins attenuates radiation-induced lung fibrosis

**DOI:** 10.1038/s41598-018-19343-9

**Published:** 2018-01-17

**Authors:** Jian Wang, Fangzheng Zhou, Zhenyu Li, Hong Mei, Ye Wang, Hong Ma, Liangliang Shi, Ai Huang, Tao Zhang, Zhenyu Lin, Gang Wu

**Affiliations:** 1 Cancer Center, Union Hospital, Tongji Medical College, Huazhong University of Science and Technology, Wuhan, 430022 China; 2 Department of Pediatric Surgery, Union Hospital, Tongji Medical College, Huazhong University of Science and Technology, Wuhan, 430022 China

## Abstract

Radiation-induced lung injury has restricted radiotherapy for thoracic cancer. The purpose of this study was to investigate the radioprotective effects of bromodomain and extra terminal (BET) inhibitor JQ1 in a murine model of pulmonary damage. Chest computed tomography (CT) was performed in a rat model after 20 Gy radiation of the right thorax. And histological evaluation and protein expressions of irradiated tissue were analyzed to confirm the potential anti-fibrosis effect of JQ1 and its underlying mechanisms. Moreover, colony formation assays were used to explore the effects of JQ1 on esophageal cancer Eca109 and breast cancer MCF7. JQ1 attenuated radiologic and histologic presentations of radiation-induced fibrosis, inflammatory reaction and pulmonary structural changes and the increase of Hounsfield units (HU) density and hydroxyproline content after radiation. Additionally, JQ1 suppressed BRD4, c-MYC, Collagen I, TGF-β, p-NF-κB p65, p-Smad2 and p-Smad3 expressions after irradiation, repressed proliferation and transdifferentiation of lung fibroblasts, and impaired clonogenic survival of thoracic cancer cells. Collectively, our study demonstrated for the first time that BET Bromodomain inhibitor JQ1 protected normal lung tissue after radiation, and exerted a radiosensitizing effect in thoracic cancer cells.

## Introduction

Radiotherapy plays an important role in the treatment of thoracic malignancies including esophageal cancer, breast cancer and non-small cell lung cancer. However, radiation doses delivered is usually restricted because of the increased risk of radiation-induced lung fibrosis (RILF)^1,2^.The presentation of radiation induced damage includes acute reactions like pneumonitis and chronic fibrosis^3–5^. Although technological advance of radiotherapy significantly reduces side effects, there are still almost 15% of cancer patients develop radiation induced pneumonitis and fibrosis^6,7^. Although proinflammatory and proliferative responses were found to be associated with the process of radiation-induced pulmonary fibrosis, its underlying mechanisms are far from being understood^8,9^. Moreover, there is no progress in the development of effective and specific anti-fibrotic therapies for routine clinical use.

Recent studies demonstrated that radiation-induced lung fibrosis is characterized by a tissue repair response triggered by chronic inflammation^10–12^. A cascade of fibrosis-associated cytokines induced by radiation has been identified, including fibrogenic TNF-α, TGF-β, IL-1 *et al*.^13–15^. More recently, molecular advances on radiation-induced response and fibrogenesis showed an essential role of epigenetic regulation^16,17^. The BET family proteins, including four members (BRD2, BRD3, BRD4, and bromodomain testis specific protein), are epigenetic reader proteins that specifically recognize acetylated chromatin and involved in regulating gene expressions. These epigenetic readers act as scaffolds and attract components of the transcriptional machinery to these acetylated lysine residues, resulting in modulation of gene transcription^18,19^. Directly or through recruitment of other transcriptional regulators, these proteins play critical roles in the control of diverse biological processes, including inflammation, cell cycle, maintenance of higher-order chromatin structure and DNA damage signaling^20–22^. The recent discovery of selective small molecule antagonists of BRD4 has introduced the possibility of exploring involvement of these epigenetic readers in a wider range of indications. Recently, targeting the BET proteins via inhibitors like JQ1 has emerged as a novel approach to treat fibrosis^23–25^.

In this study, we evaluated the radioprotective effects of a small-molecule inhibitor of BET bromodomains, JQ1 in a rat model and also investigated whether its effect was selectively delivered in tumor tissues. Weight loss was assessed and CT scan was performed after 20 Gy irradiation at one fraction for the whole right thorax. In addition, lung fibrosis was evaluated and protein expressions of right lungs were analyzed to confirm the potential anti-fibrosis effect of JQ1 and its underlying mechanisms. Moreover, the effects of JQ1 on irradiated normal human lung fibroblasts cells and thoracic cancer cells including Eca109 and MCF7 cells were also evaluated.

## Results

### CT imaging of rat lungs after irradiation

**Figure 1:**
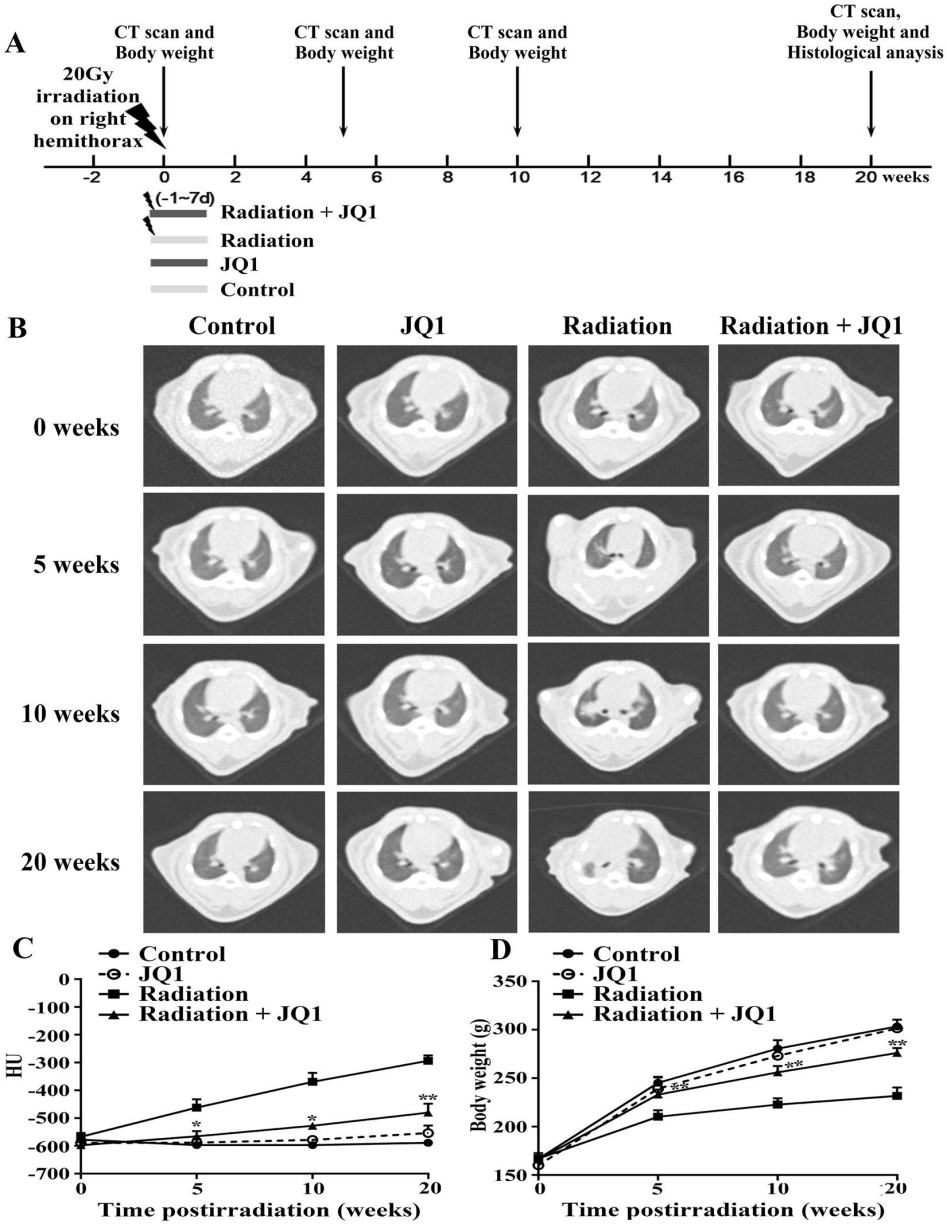
Treatment schedule and CT measurement of rat lungs after thoracic irradiation. (**A**) Rats were irradiated with a dose of 20 Gy to the right thorax at time point zero to initiate pulmonary fibrosis and were treated with JQ1 or vehicle for 9 days 24 h before the irradiation. Sham-treated rats were served as controls. Rats were monitored by histology and by noninvasive radiological methods allowing for longitudinal analysis. (**B**) CT was used for evaluation of fibrosis. Representative CT imaging showed progression of lung fibrosis in rats after 20 Gy right hemithoracic radiation with or without JQ1. (**C**) Quantitative lung density values were defined in Hounsfield Units (HU) (n = 3). Mean ± SE are presented. (**D**) Changes of animal body weight after radiation and JQ1treatment (n = 3). Mean ± SD are presented. *P < 0.05 vs. Radiation group, **P < 0.01 vs. Radiation group.

The rats were divided into four experimental groups randomly: Control group, JQ1 group, Radiation group and Radiation + JQ1 group. According to the literature and to pilot experiments performed, the rats in Radiation and Radiation + JQ1 group were treated with right thorax irradiation at a dose of 20 Gy only once using a linear accelerator by 6 MV X-rays and the rats in the Control group and JQ1 group received sham irradiation (0 Gy) of the right thorax under the same conditions. JQ1 was started 24 h before radiation and given intraperitoneally route at 50 mg/kg or vehicle daily for 9 days duration. Animals were followed until 20 weeks after the right thorax irradiation. After 10 weeks, the irradiated lungs presented with typical radiological signs of fibrosis, including septal thickening and patchy peripheral reticular abnormalities (Fig. 1B). At weeks 20 after radiation, signs of fibrosis turned out to be more obvious, indicating a progressive course of radiation-induced fibrosis. Moreover, JQ1 significantly diminished the increase of lung density after radiation at all-time points analyzed (*P* < 0.05; Fig. 1C). Thoracic radiation also induces body weight loss, which was partially attenuated by JQ1 (*P* < 0.05; Fig. 1D).

### Histological evaluation of fibrosis after irradiation

**Figure 2:**
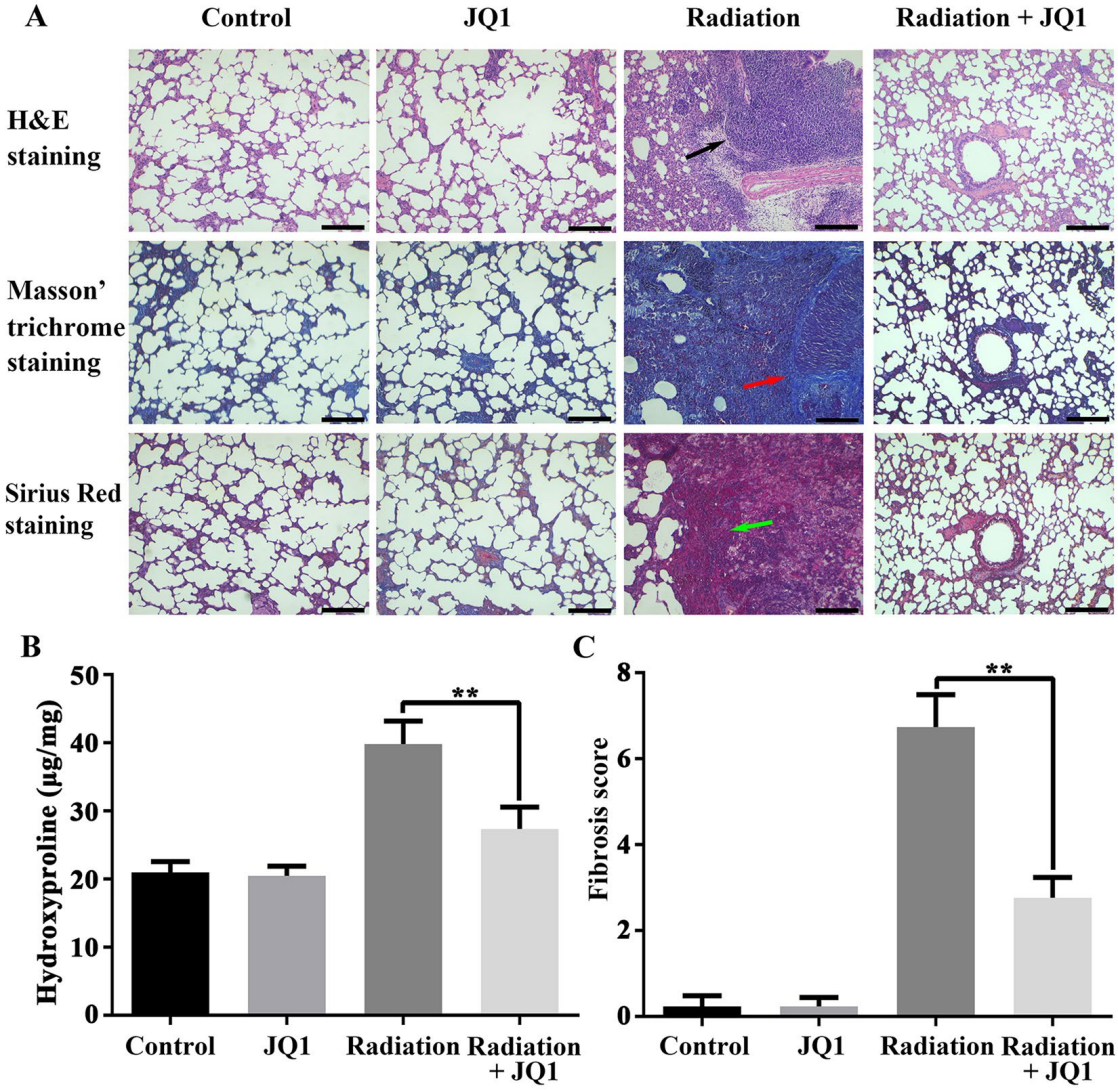
JQ1 attenuated radiation-induced histological changes of pulmonary injury. (**A**) Hematoxylin-eosin, Masson’s trichrome and Sirius red staining of right lungs excised from rats treated with vehicle, JQ1, 20 Gy irradiation, or irradiation + JQ1. Images (Scale bars, 250 μm) were typical and representative of each study group (Inflammatory cells: black arrow; collagen depositions: red arrow, green arrow) (n = 3). (**B**) Hydroxyproline content in right lung tissue of four experimental groups. Three samples from three different rats in each group. (**C)** Fibrosis scores for grading lung histopathological changes. Results were shown as the means ± SD. *P < 0.05 vs. Radiation group.

To further clarify the mechanism of the radiation-induced fibrosis, and to assess the radioprotective role of JQ1, histological analysis of right lungs in each group were further applied. Hematoxylin and eosin staining demonstrated the presence of interstitial septal thickening, inflammatory infiltration, fibrotic nodules, and alterations of alveolar structures after radiation. And JQ1 significantly attenuated the histological changes of alveolar structures and pulmonary parenchyma (Fig. 2A). The Masson’s trichrome and Sirius red staining demonstrated a lower level of collagen deposition in Radiation + JQ1 group as compared to the Radiation group. The black arrow indicates presence of inflammatory cells, while red and green arrow both means the presence of collagen depositions. Moreover, hydroxyproline content of the right lung tissue (three samples from three different rats in each group at 20 weeks after irradiation) after radiation and JQ1 combined treatment was lower than that of the rats in Radiation group (*P* < 0.05; Fig. 2B). Additionally, the fibrosis score, quantitation of the assessment of lung fibrosis, further confirmed the decreased fibrosis after JQ1 treatment in the irradiated animals (*P* < 0.05; Fig. 2C).

### Molecular analysis of lung fibrosis after irradiation

**Figure 3:**
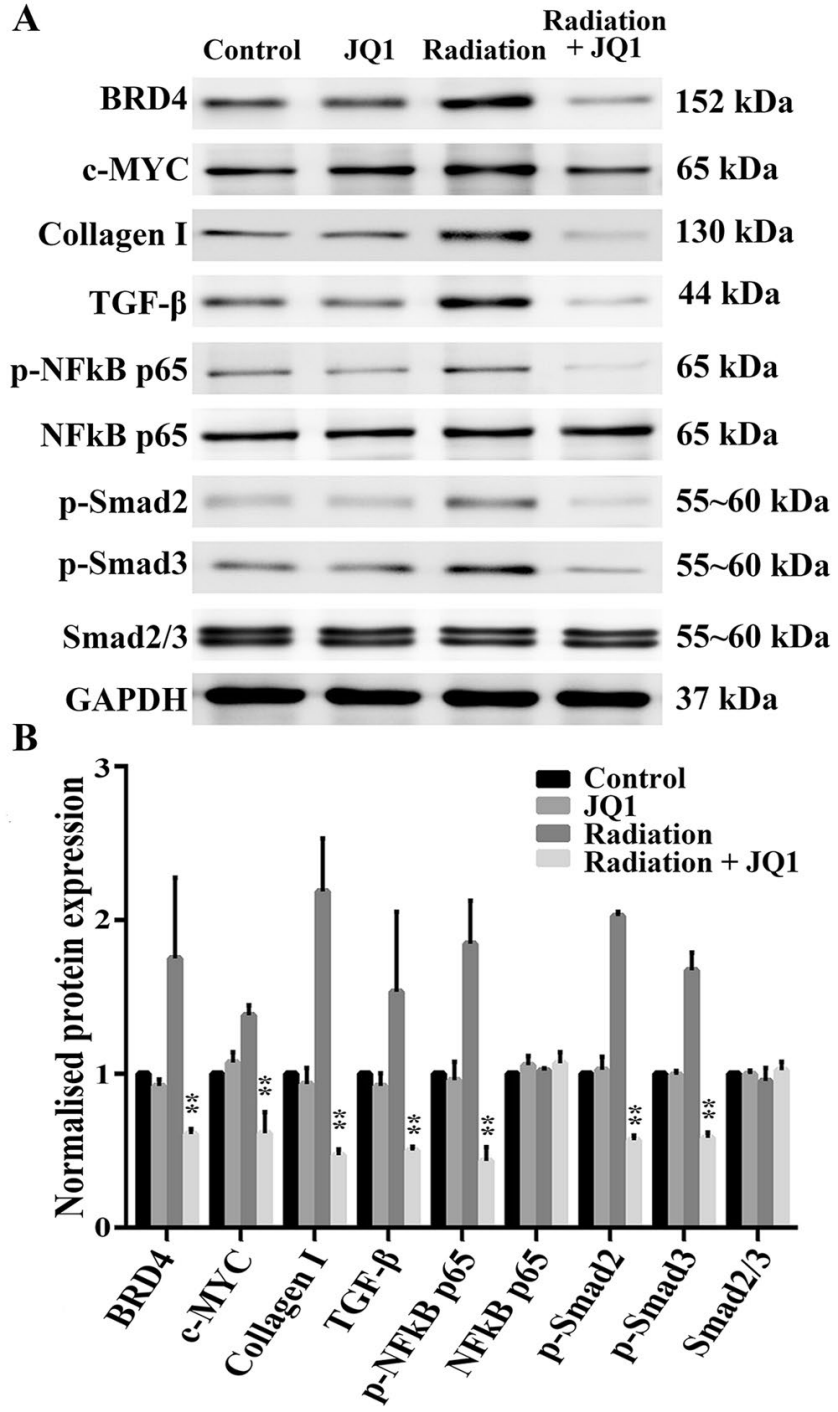
JQ1 diminished radiation-induced expressions of fibrosis markers. (**A**) Representative western blot pictures of BRD4, c-MYC, Collagen I, TGF-β, p-NFκB p65, NFκB p65, p-Smad2 and p-Smad3 in right lung tissues. Three samples from three different rats in each group were analyzed at 20 weeks postirradiation. GAPDH was used as a loading control. (**B**) Densitometric evaluation of protein bands normalized for GAPDH. Data were shown as the mean ± SD. *P < 0.05 vs. Radiation group, **P < 0.01 vs. Radiation group.

JQ1, a highly selective BET bromodomain inhibition, has displayed broad antineoplastic effects in a range of tumors carrying different genetic lesions. Proteins were extracted from the lung (three samples from three different rats in each group) 20 weeks postirradiation. As shown in Fig. 3A and B, treatment with JQ1 and 20 Gy X-ray reduced the expression levels of BRD4 and c-MYC in lung tissues of rats compared with 20 Gy X-ray alone (*P* < 0.05). To further evaluate the mechanism of radioprotective role of JQ1, western blot was performed to evaluate changes of fibrosis and inflammatory markers. JQ1 was able to significantly decreased expressions of Collagen I, TGF-β and phosphorylation of NF-κB p65, p-Smad2 and p-Smad3 increased by irradiation (*P* < 0.05), indicating its potential role in preventing radiation-induced fibrosis.

### JQ1 repressed proliferation and transdifferentiation of lung fibroblasts postirradiation

**Figure 4:**
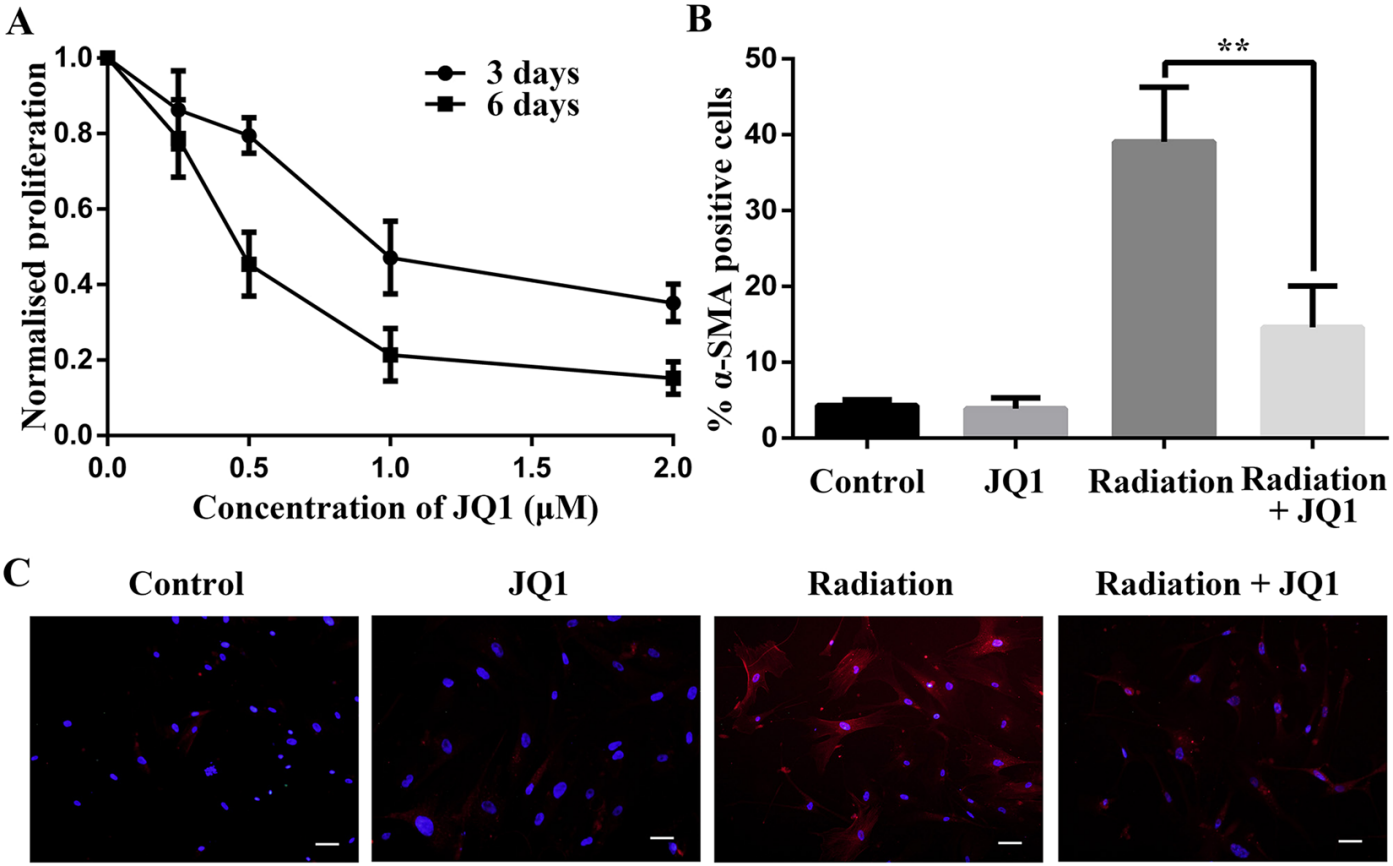
JQ1 inhibited proliferation and transdifferentiation of lung fibroblasts postirradiation. (**A**) Cell viability of 10 Gy-irradiated NHLF treated with different concentrations of JQ1 for indicated time. Cell proliferation was normalized to the irradiated NHLF without JQ1 treatment. (**B**) Inhibitory effect of JQ1 on irradiation induced α-SMA expression in NHLF cells. JQ1 pretreated NHLF cells were irradiated at the dose of 10 Gy, cultured for 6 d, and fixed. Immunofluorescence staining of α-SMA was performed as described in Materials and Methods. (**C**). Representative immunofluorescence images for B (Scale bars, 100 μm). Results were shown as the means ± SD. **P < 0.01 vs. Radiation group.

As shown in CCK-8 assay (Fig. 4A), JQ1 suppressed proliferation of normal human lung fibroblasts (NHLF) after 10 Gy X-ray irradiation in a dose- and time- dependent manner. It is known that transdifferentiation of fibroblasts to myofibroblasts by TGF-β1 boosts collagen synthesis^26^. We next explored the role of JQ1 on transdifferentiation of NHLF postirradiation by detecting the expression of α-SMA. And immunofluorescence staining assay displayed that increased expression of α-SMA by irradiation was partially suppressed by JQ1 (*P* < 0.05; Fig. 4B and C).

### JQ1 radiosensitized thoracic cancer cells

**Figure 5:**
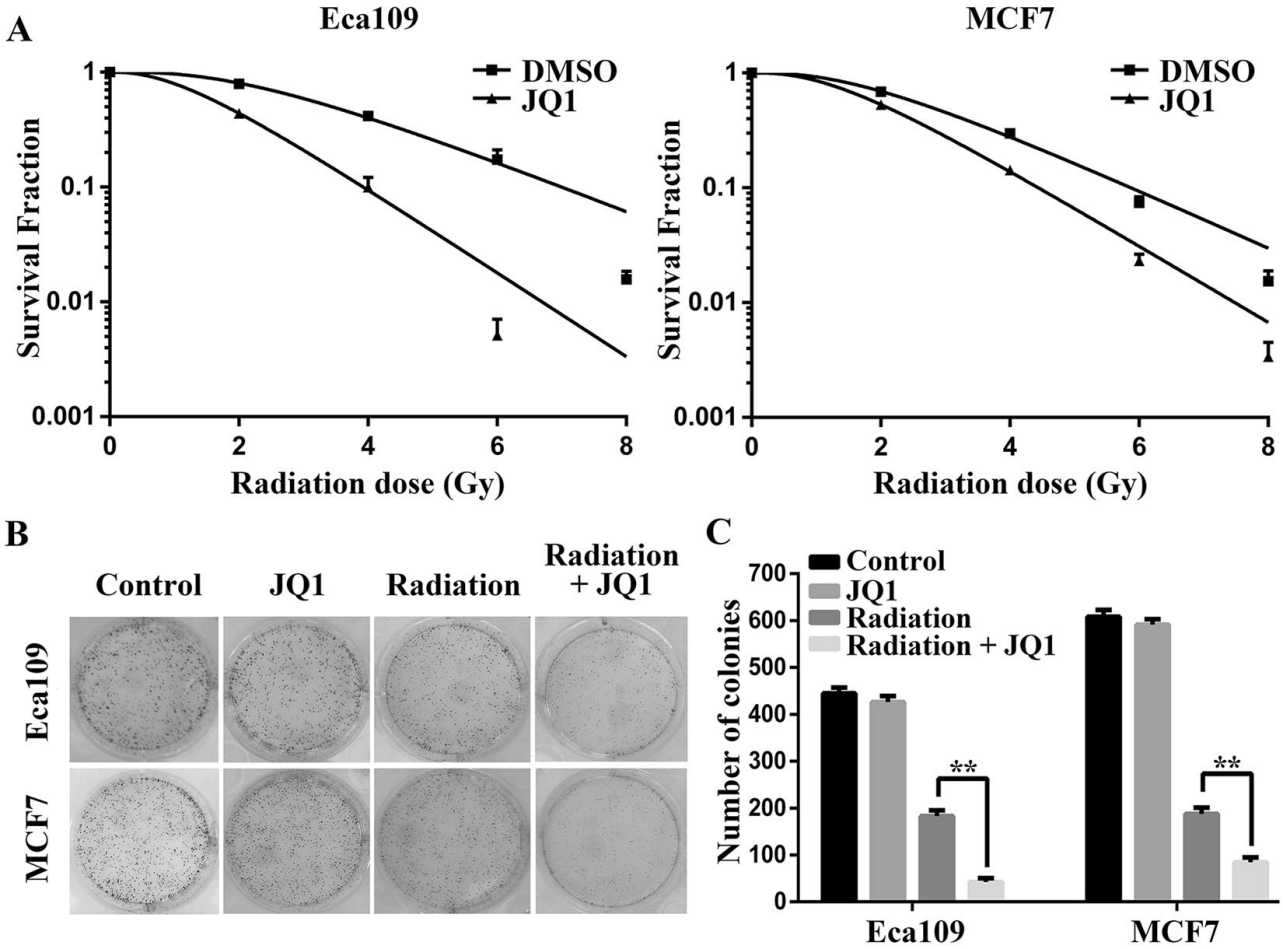
JQ1 radiosensitized esophageal cancer and breast cancer cells *in vitro*. (**A**) Colony formation assay was performed as described in Materials and Methods. (**B**). Representative images of colony formation in esophageal cancer Eca109 and breast cancer MCF7 cells treated with 4 Gy irradiation alone or combined with JQ1 (500 nM). (**C**) The numbers of colonies of Eca109 and MCF7 cells treated with radiation alone or with JQ1. Results were shown as the means ± SD at least three independent experiments. **P < 0.01 vs. Radiation group.

A good radioprotective agent should not only be protective in normal tissues, but also not induce cancer cell progression. Our previous study illustrated that JQ1 radiosensitizes non-small cell lung cancer cells (NSCLC) *in vitro* and *in vivo*^27^. Next, we detected the radiosensitivity of the Eca109 and MCF7 cells treated with or without JQ1 by colony formation assay (Fig. 5A). The radiosensitive parameters were shown in Table 1. The values of SF_2_, D_0_, D_q_ and N decreased after treated with JQ1, indicating the increased radiosensitivity of Eca109 and MCF7 cells. The sensitization enhancement ratio (SER) of Eca109 and MCF7 cells was 1.81 and 1.29 respectively. Meanwhile, JQ1 significantly reduced the surviving colonies (with more than 50 cells per colony) of irradiated Eca109 and MCF7 cells (*P* < 0.05; Fig. 5B and C). Collectively, these results suggested that JQ1 enhanced the radiation response of thoracic malignancies including NSCLC, esophageal cancer and breast cancer.

**Table 1:** The radiosensitive parameters of Eca109 and MCF7 cells treated with or without JQ1.

Parameter	Eca109		MCF7	
	DMSO	JQ1	DMSO	JQ1
D_0_	1.97	1.19	1.53	1.30
D_Q_	5.19	2.16	4.07	2.76
SF_2_	0.79	0.44	0.69	0.53
N	3.63	2.82	3.65	3.12
SER		1.81		1.29

D_0_, mean lethal dose; D_q_, quasi-threshold dose; SF_2_, surviving fraction at 2 Gy; N, extrapolation number; SER, sensitization enhancement ratio.

## Discussion

Radiation-induced lung fibrosis is a severe side effect of radiotherapy in the management of thoracic cancer and it significantly impacts life quality of cancer patients. Clarifying the underlying mechanisms of RILF is vital for the development of effective strategies. Several recent studies have implicated BRD4 in the development of experimental models of sepsis, multiple myeloma, and chronic kidney disease. Herein we have demonstrated for the first time that pharmacologic inhibition of BRD4 by JQ1 was safe and feasible, but also significantly attenuated radiologic and pathologic presentations of RILF as well. These findings indicate that BET bromodomains are critically involved in the activation of profibrotic machinery in the lung, and suggest that pharmacological targeting of BET proteins could be a therapeutic option for RILF treatment.

Minimizing radiation-induced lung injury is an important goal of therapy. RILF is similar to other forms of pulmonary fibrosis, either of iatrogenic origin such as from chemotherapy or surgery or idiopathics^28^. Irrespective of the initial cause, fibroblast replication with excessive extracellular matrix deposition is the hallmark of the disease. The clinical manifestation includes progressive dyspnea, deterioration of pulmonary function, and interstitial fluid accumulation resulting in respiratory failure. Although modern radiotherapy techniques are increasingly applied to improve dose distribution, radiation induced fibrosis was inevitable in clinical due to the variation of normal tissue tolerance^10,29^. Consequently, the development of effective antifibrotic therapy is urgent to enhance the efficacy of radiotherapy.

BET family members are a cluster of proteins that regulate gene transcription via recognizing acetylated histones and subsequently recruiting transcription factors. Recently many inhibitors targeting BET proteins were developed. JQ1 is a small-molecule BET bromodomain inhibitor, which mainly competitively occupies the recognition site of BRD4^19^. And it was found to be highly efficient in inhibiting a variety of cancer cells and tumor growth^18,30,31^. Moreover, recent studies revealed a wider prospective application of BET inhibitors in preventing fibrosis^32^, septic shock^33^, and even innate immunity^34^. For example, Budd *et al*. demonstrated that inhibition of BRD4 by JQ1 can block TGF-β1 and PDGF-induced migration and proliferation of fibroblasts isolated from lung tissues of patients with idiopathic pulmonary fibrosis^35^. Oral administration of JQ1 was also capable of preventing the development of bleomycin-induced pulmonary fibrosis in murine model^32^. In addition, Ding *et al*. found that JQ1 was also effective in blocking activation and proliferation of hepatic stellate cell activation *in vitro* and *in vivo*^23^. It is reported that BRD4 inhibition by JQ1 attenuates unilateral ureteral obstruction-induced fibrosis in a murine model^25^. To date, there are still no reports assessing the pharmacological effect of BET inhibitors on radiation-induced lung fibrosis. Consequently, we assessed whether this drug could be potentially efficient in minimizing RILF, based on its robust effects on gene regulation. Our data showed that JQ1significantly alleviated the presentation of radiation-induced pulmonary injury in rats, as demonstrated by CT films and histological presentations of the lungs, indicating its potential radioprotective role.

Although the underlying mechanism of RLIF is still not clear, accumulating evidence demonstrates that a variety of cytokines play important roles in the tissue reorganization and immune response in RILF. In this study, the radioprotective effect of JQ1 was further confirmed by the decreased expression of radiation-induced collagen, and BET inhibition via JQ1 was able to block activation of multiple intracellular signaling pathways associated with radiation-induced fibrosis. The cytokine TGF-β is known as an important regulator of cell growth and differentiation expressed in response to injuries induced normal tissue damage, especially in RILF^36–38^. TGF-β was upregulated in mouse lungs hours to weeks after radiation^39^. TGF-β transduced its fibrotic signal through activation (phosphorylation) of Smad-2 and Smad-3^8^. Thus, we evaluated the influence of JQ1 on the expressions of TGF-β, phosphorylation of Smad2 and Smad3 and demonstrated that JQ1 treatment inhibited radiation-induced Smad2 and Smad3 phosphorylation. In addition, we found that JQ1 inhibited activation of NF-κB, another pathways involved in the regulation of proinflammatory responses. Therefore, it appears that JQ1 can attenuate radiation-induce fibrosis through suppressing activation of multiple intracellular signaling pathways associated with lung fibroblast activation and proinflammatory responses. Moreover, JQ1 inhibited proliferation and transdifferentiation of irradiated normal human lung fibroblasts. However, the BET-targeted gene expression profiles and the precise molecular mechanisms mediating the radioprotecting effects of BET inhibitors remain to be defined due to its broad biological effects.

A good radioprotective agent should not only be protective in normal tissues, but also not induce cancer cell progression. The potential antitumorigenic effects of JQ1 have been studied in numerous *in vitro* and *in vivo* studies on several cancer models^18,19,40^. To confirm whether the radioprotective role of JQ1 was selective for normal tissues alone, we further performed clonogenic assays. No protection of Eca109 and MCF7 cells upon JQ1 treatment was observed. Conversely, JQ1 plus irradiation significantly reduced surviving colonies compared to irradiation alone.

In conclusion, our study demonstrated that JQ1 protected normal lung tissue after radiation, but also exerted a radiosensitizing effect in thoracic cancer cells including NSCLC^27^, esophageal cancer and breast cancer cells. Although further research is required, we present the first evidence for the application of JQ1 as a radioprotective agent of lungs in the radiotherapy of thoracic malignancies.

## Materials and Methods

### Cell culture and reagents

Eca109 (esophageal cancer), MCF7 (breast cancer) and normal human lung fibroblasts (NHLF) cells were cultured according to ATCC. JQ1 was purchased from Selleckchem and formulated in DMSO. The following primary antibodies were used: BRD4 (WB:1:1000 dilution, Abcam), c-MYC (WB: 1:200 dilution, Santa Cruz), Collagen 1a (Col1) (WB: 1:1000 dilution, Abcam), TGF-β (WB: 2 μg/ml, Biorbyt), p-NFκB p65 (ser 276; WB: 1:500 dilution, Santa Cruz), NFκB p65 (WB: 1:500 dilution, SantaCruz), p-Smad2 (Ser465/467) (WB: 1:1000 dilution, CST), p-Smad3 (Ser423/425) (WB: 1:1000 dilution, CST), Smad2/3 (WB: 1:500 dilution, Santa Cruz), and GAPDH (WB: 1:3000 dilution, Abcam), α-smooth muscle actin (α-SMA) (IF: 1:500; Abcam).

### Murine model of pulmonary fibrosis

Female SD rats (Beijing HFK Bioscience, China) aged 7–8 weeks and weighing 150–170 g were used. In the main experiment, we used computer-generated random numbers to divide the rats into four experimental groups: Control group (*n* = 3), JQ1 group (*n* = 3), Radiation group (*n* = 3) and Radiation + JQ1 group (*n* = 3). JQ1 was started 24 h before radiation and given intraperitoneally route at 50 mg/kg or vehicle daily for 9 days duration. The rats were anesthetized intraperitoneally with pentobarbital sodium (50 mg/kg) and kept in a supine position over a specially designed recess foam bed. For right hemithoracic irradiation, thoracic regions were aligned using CT simulation images and a rectangular irradiation field with the same size of the right lung was used. According to the literature and to pilot experiments performed, the rats in Radiation group and Radiation + JQ1 group were treated with right thorax irradiation at a dose of 20 Gy only once by 6 MV X-rays. The dose rate was 600 MU/min. The rats in the Control group and JQ1 group received sham irradiation (0 Gy) of the right thorax under the same conditions. Animals were followed until 20 weeks after radiation. CT imaging and body weight evaluation were performed regularly as indicated in Fig. 1A.

### Ethics statement

All experimental protocols were approved by the Ethical Committee of Huazhong University of Science and Technology (HUST, China) and conducted in accordance with the Animal Care and Use Committee protocols of HUST and the guidelines for the welfare and use of animal in cancer research^41^.

### CT scan

High resolution computed tomography (hrCT) was performed by the CT-SIM Scanner System (Philips) as previous described^42^. In this experiment, three rats per group were received CT scan. The CT images were obtained and imported into RadiAnt DICOM Viewer 3.4.1 (http://www.radiantviewer.com/). Lung density was expressed as CT intensity units in analogy to Hounsfield units used in clinical CT scanners. Slices of lung images were made at the tracheal bifurcation. In each selected slice 2 regions of interest were defined: right anterior and right posterior. A total of 2 density values per right lung were determined and the arithmetic mean ± SE defined the representative CT intensity.

### Lung histology and fibrosis score

Twenty weeks postirradiation, rats were sacrificed and the right lung tissues were removed. Parts of upper lung lobes (three samples from three different rats in each group) were dried and subjected to acid hydrolysis according to the protocol in the Hydroxyproline Testing Kit. The rest of the right lungs were prepared for other analysis. Lung tissues were placed in 4% paraformaldehyde for 24 hours following staining with hematoxylin-eosin (H&E), Masson’s trichrome and Sirius red. Grading criteria of pulmonary fibrosis was classified into 9 grades by two blinded observers independently according to the grade of lung fibrosis scored on a scale from 0 to 8 by examining random sections at 100× magnification^43^. An average score was determined from five randomly selected microscopic fields in each section (three samples from three different rats in each group).

### Western blot analysis

Proteins of lung tissues were extracted by a protein extraction kit (Pierce Biotechnology Inc.) 20 weeks postirradiation (three samples from three different rats in each group). Concentration was determined by the use of bicinchoninic acid (BCA) assay. Proteins were submitted to 10% polyacrylamide SDS gel electrophoresis and transferred to a nitrocellulose membrane. And it was blocked with 5% nonfat skim milk in Tris-buffered saline Tween (TBST) buffer for 2 h, following incubating with primary overnight at 4 °C and subsequent secondary antibodies for 1 h at RT. Image J software was applied for densitometric analysis of protein bands. GAPDH protein levels were used as a control.

### Cell viability assay and colony formation assay

The effect of JQ1 on cell viability of NHLF was determined by CCK-8 assay and colony formation of MCF-7 and ECA109 was performed as previously described^27^. Cell suspensions were diluted serially to appropriate densities depending on the exposure dose and plated on six-well plates in triplicate. After attachment, the cells were treated with DMSO or 500 nM JQ1 and then after 24 h of incubation, exposed to different doses of radiation respectively. 48 h after irradiation, cells were cultured in drug free medium for 10 to 14 days and then stained with 0.5% crystal violet (Sigma). Plating efficiency was calculated as a ratio of the number of colonies to the seeding cells without radiation. The surviving fraction at each radiation dose was calculated as number of colonies/(number of seeding cells’ plating efficiency). The survival curve was fit into a multi-target single-hit model, and radiobiological parameters were calculated including surviving fraction at 2 Gy (SF_2_), mean lethal dose (D_0_), quasi-threshold dose (D_q_), extrapolation number (N), and sensitization enhancement ratio (SER).

### Immunofluorescence Staining

Staining was performed and analyzed as previously described with modifications^44^. Cells were divided into four groups: Control (DMSO), JQ1 (500 nM), Radiation (8 Gy), Radiation + JQ1 group. After attachment, the cells were pretreated with 500 nM JQ1 or DMSO for 24 h and irradiated at a single dose of 8 Gy. At 6 days after irradiation, cells were fixed with 4% polyformaldehyde and stained with rabbit α-SMA antibody followed by cy3-conjugated secondary antibody (1:300; ABclonal). Nuclear was counterstained with DAPI (Sigma). Five high-power fields per slide were counted to calculate the percentage of α-SMA-positive cells of total cells.

### Statistics

Statistical analysis was performed using GraphPad Prism software. Student’s t test was applied, and Probability values were considered significant at *P* < 0.05. Data are expressed as the mean ± SD or mean ± SE.

## Electronic supplementary material

Supplementary figure
