# Supplementary material for: Pharmacological targeting of BET proteins attenuates radiation-induced lung fibrosis
Source: Sci Rep. 2018 Jan 17;8:998. doi: 10.1038/s41598-018-19343-9 (PMC5772369; doi:10.1038/s41598-018-19343-9)
Supplement: Supplementary file 1 — Supplementary figure [file 41598_2018_19343_MOESM1_ESM.pdf]

Pharmacological targeting of BET proteins attenuates radiation-induced lung fibrosis

Jian Wang [a, #], Fangzheng Zhou [a, #], Zhenyu Li [a], Hong Mei [b], Ye Wang [a], Hong Ma [a], Liangliang Shi [a], Ai Huang [a], Tao Zhang [a], Zhenyu Lin [a, *], Gang Wu [a, *]

[a] Cancer Center, [b] Department of Pediatric Surgery, Union Hospital, Tongji Medical College, Huazhong University of Science and Technology, 1277 JieFang Avenue, Wuhan 430022, China

[#] Both authors contributed equally to this work.

[*] These authors jointly directed this work. Correspondence should be addressed to Zhenyu Lin (unionlzy@126.com) or Gang Wu (xhzlwg@163.com)

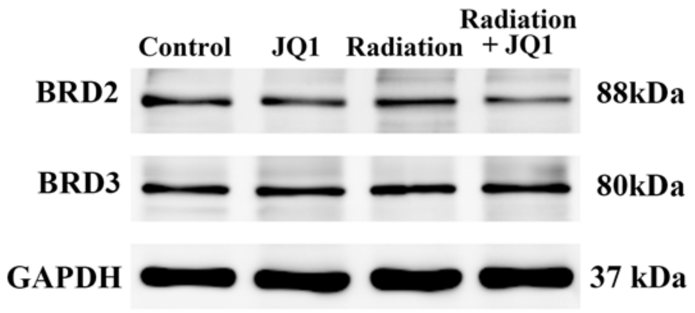

Figure S1：Expressions of BRD2 and BRD3 in right lung tissues from each group were analyzed at 20 weeks postirradiation. GAPDH was used as a loading control.
